# Deaths from Bacterial Pneumonia during 1918–19 Influenza Pandemic

**DOI:** 10.3201/eid1408.071313

**Published:** 2008-08

**Authors:** John F. Brundage, G. Dennis Shanks

**Affiliations:** * Armed Forces Health Surveillance Center, Silver Spring, Maryland, USA; † Australian Army Malaria Institute, Enoggera, Queensland, Australia

**Keywords:** Influenza, pandemic, epidemiology, bacterial pneumonia, opportunistic infections, mass immunization, history, public health practice, infection control, historical review

## Abstract

Deaths during the 1918–19 influenza pandemic have been attributed to a hypervirulent influenza strain. Hence, preparations for the next pandemic focus almost exclusively on vaccine prevention and antiviral treatment for infections with a novel influenza strain. However, we hypothesize that infections with the pandemic strain generally caused self-limited (rarely fatal) illnesses that enabled colonizing strains of bacteria to produce highly lethal pneumonias. This sequential-infection hypothesis is consistent with characteristics of the 1918–19 pandemic, contemporaneous expert opinion, and current knowledge regarding the pathophysiologic effects of influenza viruses and their interactions with respiratory bacteria. This hypothesis suggests opportunities for prevention and treatment during the next pandemic (e.g., with bacterial vaccines and antimicrobial drugs), particularly if a pandemic strain–specific vaccine is unavailable or inaccessible to isolated, crowded, or medically underserved populations.

## Abstract 2

A sequential-infection hypothesis is consistent with characteristics of this pandemic.

Many influenza experts, policy makers, and knowledgeable observers believe that a novel influenza A (H1N1) strain directly caused most deaths during the 1918–19 pandemic, often from a hemorrhagic pneumonitis that rapidly progressed to acute respiratory distress syndrome and death (*1**–**3*). Not surprisingly, plans and resources to respond to the next influenza pandemic focus almost exclusively on the virus, i.e., preventive vaccines and antiviral treatment of infections with a novel influenza strain (*4*). However, healthcare providers, medical experts, and published data from the 1918 period suggest that most deaths were caused by secondary bacterial pneumonias (*5**–**12*); hemorrhagic pneumonitis that rapidly progressed to death was considered an alarming but uncommon clinical manifestation (*8*,*11*–*13*).

Undoubtedly, the 1918–19 pandemic strain of influenza had unique pathophysiologic effects. In the wake of its worldwide spread, the number of deaths was unprecedented. However, contemporaneous reports suggest that the pathophysiologic effects of the virus, in and of themselves, did not directly cause most (or even many) of the deaths during the pandemic. If the pandemic strain was not inherently hypervirulent (i.e., if direct pathophysiologic effects of the virus were necessary but not sufficient to cause death in a large proportion of immunologically susceptible hosts) and if bacterial infections were also necessary causes of most deaths during the pandemic, then preparations for the next pandemic should focus on more than preventing and treating infections with a novel influenza strain alone.

We have identified epidemiologic and clinical characteristics of the 1918–19 pandemic that are not readily consistent with the view that most deaths were caused by the direct effects of an inherently hypervirulent virus and were clinically expressed as rapidly progressing, ultimately fatal pneumonitis. Our alternative hypothesis is consistent with known characteristics and firsthand accounts of the pandemic and contains implications for preparing for the next pandemic.

## Epidemiologic and Clinical Characteristics of 1918–19 Pandemic

### Disease Usually Mild and Self-limited

The 1918–19 pandemic spread worldwide with remarkable speed. Over several months, a novel strain of influenza virus attacked communities worldwide; most persons were immunologically susceptible. However, most cases followed a mild or self-limited course. Had the pandemic strain been inherently hypervirulent, in the absence of modern lifesaving measures one would expect exceptionally high case-fatality rates for all affected populations. Yet during that pandemic, most infected persons had self-limited clinical courses and complete recovery (*3*,*7*,*8*,*11*,*14*). For most affected populations, the case-fatality incidence was <2% and the overall mortality rate was <0.5% (*3*,*7*,*8*,*13*,*15*,*16*).

### Clinical Courses of Fatal Cases Highly Variable and Often Prolonged

**Figure 1:**
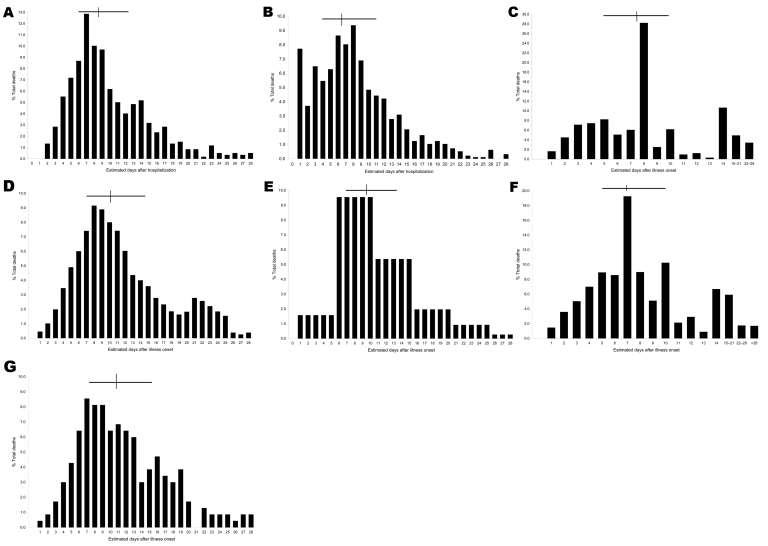
Percentage distributions of fatal cases of influenza–pneumonia during 1918–19 influenza pandemics, by estimated days of illness before death. A) Influenza–bronchopneumonia, Cook County Hospital, Chicago, Illinois, USA (n = 599) (estimated from chart 2 in [*19*]). B) Australian Imperial Forces, 1918 (n = 972) (G.D. Shanks, unpub. data). C) General population, Prussia (n = 6,223) (*22*). D) US Army autopsy series (n = 94) (estimated from supplementary Figure 2 in [*17*]). E) Influenza with secondary staphylococcal pneumonias, Fort Jackson, South Carolina, USA (n = 153) (interpolation of data in Table 1 in [*21*]). F) New South Wales, Australia (n = 3,866) (*20*). G) US Army training camp, Camp Pike, Arkansas, USA (n = 234) (*5*). Horizontal bars indicate interquartile ranges; vertical lines indicate medians.

**Figure 2:**
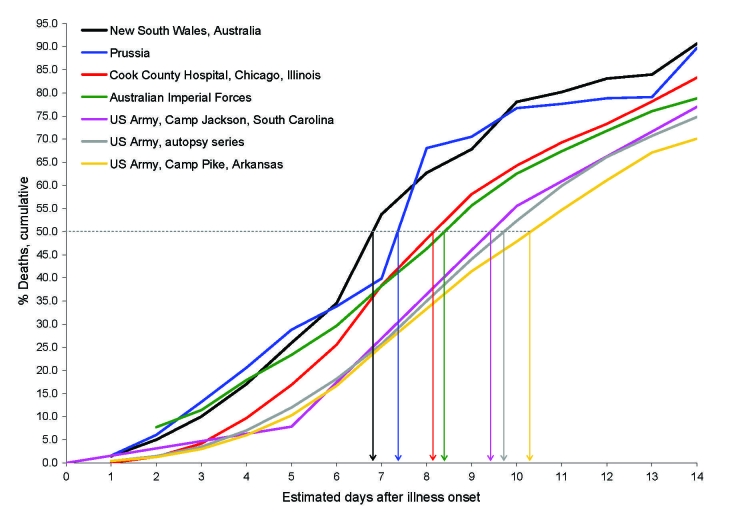
Cumulative percentage deaths from influenza–pneumonia, by days (estimated) from illness onset, among fatal cases during various epidemics, 1918–19 (*5*,*17*–*22*). Vertical arrows indicate median no. days to death.

In most affected populations, <5% of deaths occurred within 3 days of illness onset, median time from illness onset to death was 7–10 days, and significant numbers of deaths occurred >2 weeks after initial symptoms (*5*,*17*–*22*; Figures 1, 2). These findings do not suggest that an inherently virulent virus caused fulminant disease and rapid progression to death in high proportions of infected persons—or even in most fatal cases. In the prominently cited experience of Sydney, Australia, most influenza-related deaths occurred within 3 days of hospital admission (*2*,*23*,*24*); however, only the sickest patients were admitted to Sydney hospitals (*23*). In New South Wales overall, only ≈10% of fatalities occurred within 3 days of illness onset (Figure 1, panel **F**; Figure 2) (*20*).

### Progression to Death, No Difference between Early and Late Pneumonias

**Figure 3:**
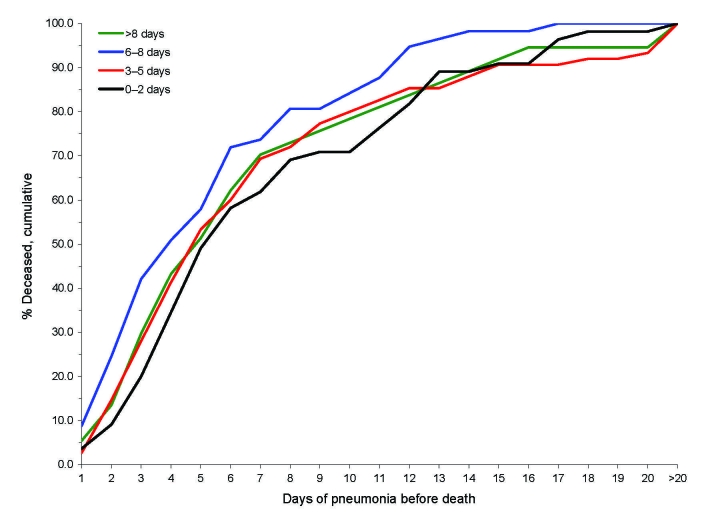
Cumulative percentage deaths by days of pneumonia, in relation to days of illness before pneumonia, among 234 US Army soldiers who died of influenza–pneumonia at Camp Pike, Arkansas, USA, autumn 1918 (*5*).

If most deaths resulted from primary influenza pneumonias that progressed rapidly, one might expect that fatal pneumonias that developed early in clinical courses would progress more rapidly than those that developed later. However, the findings of Opie et al. suggest that primary influenza pneumonias did not progress unusually rapidly to death. Opie et al. conducted postmortem examinations and documented the clinical courses of 234 fatal cases that occurred during the epidemic at Camp Pike, Arkansas, USA (*5*). They found that the durations of pneumonia before death were similar among those in whom pneumonia developed early (0–2 days) versus later (3–5, 6–8, >8 days) after influenza onset (Figure 3) (*5*).

### Mortality and Case-Fatality Rates High for Young Adults and Other Unlikely Groups

During the pandemic, overall mortality and case-fatality rates were higher for young adults, indigenous and other relatively closed populations, and certain military and occupational subgroups than for their respective counterparts. Case-fatality and mortality rates were higher for those 25–40 years of age (particularly men) than for those younger or older (*15*,*16*). Explanations have included aberrant host immune responses to infections with the subtype H1N1 pandemic strain—increasing the risk for “cytokine storm” (*1*)—and higher cardiac stroke volumes in young adults (*24*).

**Figure 4:**
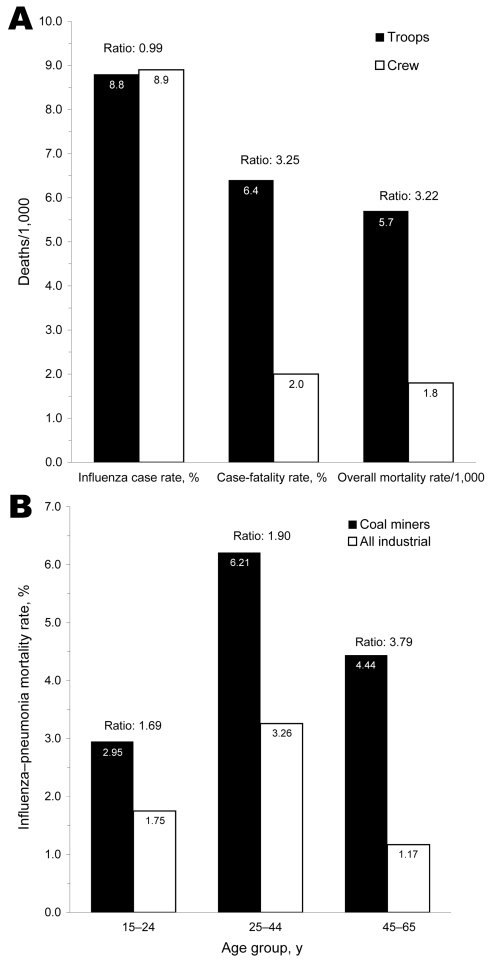
A) Influenza–pneumonia-related morbidity and mortality cumulative incidence rates, in relation to status on troop ships, Cruiser and Transport Service, US Navy, 1918 (*9*). B) Influenza–pneumonia mortality rates for white men, by employment as coal miner versus other industrial occupation, and by age group, October–December 1918 (*6*).

However, at US military training camps, recent arrivals had worse clinical outcomes than their similarly aged, male counterparts who had been in camps longer. For example, during wartime, 60% of all influenza–pneumonia deaths affected soldiers who had been in the service <4 months (total influenza–pneumonia deaths, 34,446; deaths of soldiers with <4 months of service, 20,837) (*10*). In the Australian Imperial Forces, mortality rates differed by 50-fold across units of similarly aged soldiers in France and the United Kingdom (G.D. Shanks, unpub. data). US soldiers and Marines who were being transported on ships had similar influenza case rates but higher case-fatality rates (influenza cases 11,385, case rate 8.80/1,000, deaths 733) than the sailors who were permanently assigned to the same ships (influenza cases 2,123, case rate 8.88/1,000, deaths 42) (Figure 4, panel **A**) (*9*). Among Australians and Americans, sharply higher death rates were reported for civilian miners (*6*,*25*) and military tunnelers (G.D. Shanks, unpub. data) than for their similarly aged counterparts (Figure 4, panel **B**).

In South Africa, case-fatality rates were >2× higher for “Blacks, Indians, and Coloureds” (influenza cases 2,162,152, deaths 127,745, case-fatality rate 5.9%) than for “Whites” (influenza cases 454,653, deaths 11,726, case-fatality rate 2.6%) (*26*); and the influenza-associated mortality rate was >30× higher for Kimberley diamond miners (influenza deaths 2,564, overall mortality rate 22.4%) (*26*) than for Rand gold miners (influenza cases 61,000, deaths 1,147, case-fatality rate 1.9%, overall mortality rate 0.6%) (*26*). In Rhodesia, influenza-related mortality rate was ≈4× higher in mining compounds (9.2%) than in villages (2.3%) (among mine workers, overall influenza cases 19,471, deaths 2,851, case-fatality rate 14.6%) (*27*).

During the pandemic in New Zealand, death rates were ≈7× higher for indigenous (Maori) populations (influenza deaths 2,160, mortality rate 42.3/1,000) than for other residents (influenza-related mortality rate 4.5/1,000) (*28*). Across other South Pacific islands, death rates were generally higher for indigenous populations than for others. For example, death rates in Fiji were ≈4× higher for indigenous Fijians (influenza cases 5,154, mortality rate 5.7%) than for Europeans (influenza cases 69, mortality rate 1.4%) (*8*). In Guam, where military and indigenous populations were both located, ≈4.5% of the indigenous population, but only 1 sailor assigned to the US Naval base, died (*9*). In Saipan, “practically all of the inhabitants contracted the disease”; however, the mortality rate was reportedly sharply higher for Chamorrans (12.0%) than for Caroline Islanders (0.4%) (*29*). In Western Samoa, an estimated 22% (deaths 7,542) of the entire population died (*8*,*30*).

In various communities of Canada, Sweden, Norway, and the United States, mortality rates were estimated to be 3–70× higher for indigenous than for nonindigenous populations (*8*,*31*). Across British colonial countries of the Caribbean, the difference in mortality rates was >45-fold between the least affected (Bahamas: deaths ≈60, mortality rate ≈0.1%; Barbados: deaths ≈190, mortality rate ≈0.1%) and the most affected (Belize: deaths ≈2,000, mortality rate ≈4.6%); in general, the highest mortality rates in the Caribbean affected East Indian workers, Native Americans, and the poor (*32*). 

The findings of sharply different clinical courses and outcomes in subgroups of infected persons of similar ages, sociocultural circumstances, and prior health states belie the importance of host immune intensity and cardiac stroke volume as the definitive determinants of clinical outcomes after infection. Undoubtedly, factors other than the inherent virulence of the virus or the robustness of the host’s immune response affected the clinical expressions of influenza infections. In his classic review, E.O. Jordan concluded that “one of the chief reasons for the great variation in case-fatality in different groups is undoubtedly the nature and relative abundance of secondary invaders ... The excessively high mortality in certain army camps, on certain transports and in particular hospitals or barracks seems most readily explicable in this way” (*6*).

### Common Respiratory Bacteria Most Often Recovered from Pneumonia Patients

During the 1918–19 pandemic, the bacteria most often recovered from the sputum, lungs, and blood of pneumonia patients, alive or dead, were common colonizers of the upper respiratory tracts of healthy persons, i.e., *Hemophilus influenzae*, *Streptococcus pneumoniae*, *S. pyogenes*, and/or *Staphylococcus aureus* (*5**–**13*). During local epidemics, 1 or 2 of these species accounted for most isolates from pneumonia patients (*5**–**13*). For example, among pneumonia patients at 21 US Army camps in the autumn of 1918, *S. pneumoniae* (especially types III and IV) predominated at 12 camps, *H. influenzae* at 6, and *Streptococcus* spp. at 3 (*5*). *S. aureus* was a major cause of pneumonia among persons with fatal cases at Camp Jackson, South Carolina, USA, and Camp Syracuse, New York, USA (*5*,*12*,*21*).

The bacteria most often recovered from the lungs of patients who died were all common colonizers of the upper respiratory tracts of healthy persons. Types III and IV pneumococci (ubiquitous colonizing strains) were often recovered from the lungs of patients who died during the 1918–19 pandemic but were not considered important pathogens otherwise. Opie et al. concluded, “Every patient with influenza must be considered a potential source of pneumococcus or hemolytic streptococcus infection for his neighbor ... Every person engaged in the care of patients with respiratory diseases must also be regarded as a potential source of danger” (*5*).

### Mortality Rates More Strongly Correlated with Pneumonia Rates than with Clinical Case Rates

If the pandemic strain had been inherently hypervirulent and had directly caused most influenza-related deaths, one would expect strong correlations between clinical case rates and mortality rates across affected populations. Yet in affected communities in general, correlations were stronger between mortality and pneumonia rates than between mortality and clinical case rates (*15*,*16*).

**Figure 5:**
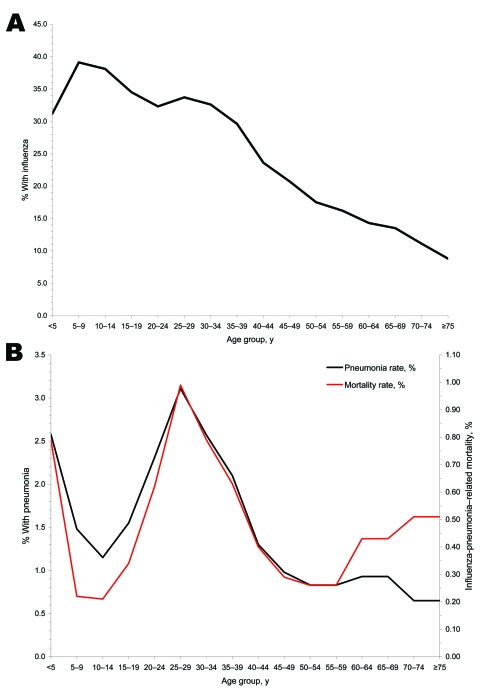
A) Estimated age group–specific influenza case rates (*15*,*16*). B) Estimated age group–specific pneumonia rates and mortality rates, based on household surveys of 10 communities throughout the United States (*15*,*16*).

In general, age-related mortality rates and pneumonia rates—but not clinical case rates—were W-shaped with sharp peaks for young adults. Influenza-related mortality rates peaked sharply for young adults 25–40 years of age. Data from household surveys throughout the United States suggest that pneumonia case rates also peaked for young adults (Figure 5) (*15*,*16*). In contrast, influenza case rates were highest for school-aged children, plateaued at a lower level for young adults, and continuously declined through older age groups (Figure 5) (*15*,*16*).

After reviewing US household survey data, a senior statistician of the US Public Health Service concluded that “... these relations indicate that the mortality is determined primarily by the incidence of pneumonia. The cause of the high mortality in young adult life evidently lies in the complicating pneumonia. All of the relations ... bear this out ...” (*16*)

### Nonpharmaceutical Interventions Associated with Lower Overall Mortality Rates

Systematic analyses of mortality data from large US cities have shown that nonpharmaceutical interventions (e.g., isolation, quarantine, closing schools, banning public gatherings) were associated with lower influenza-related mortality rates during the autumn of 1918 (*33*). Given the rapidity of spread of the pandemic, reductions of mortality rate associated with nonpharmaceutical interventions are unlikely to have been primarily related to reductions of influenza transmission (particularly in large US cities during wartime).

On the basis of their extensive studies in US Army camps during the 1918–19 pandemic, Opie et al. concluded that “Secondary contact infection may be responsible for the development of pneumonia in patients with influenza. ... It is probable that secondary contact infection can be effectively prevented only by individual isolation and strict quarantine of every patient.” (*5*) Perhaps the reduction in mortality rate after isolation, quarantine, and other social distancing measures were implemented resulted from decreased exposures of persons with influenza to bacterial respiratory pathogens to which they were transiently highly susceptible.

### Firsthand Accounts and Reviews: Most Deaths Caused by Secondary Bacterial Pneumonias

During the pandemic, medical journals contained hundreds of detailed reports of local influenza epidemics. In addition, during and after the pandemic, remarkably detailed reviews of relevant epidemiologic and clinical records and population-based surveys were conducted by government and academic institutions worldwide. Care providers and experts of the day in epidemiology, pathology, bacteriology, and infectious diseases clearly concurred that pneumonias from secondary bacterial infections caused most deaths during the pandemic (*5**–**14*). In his classic review, Jordan summarized the key factors involved in the production of influenza-related pneumonia during the pandemic as follows:

“(1) The influenza virus weakens the resistant power of the pulmonary tissues so that various bacteria are able to play the role of secondary invaders; (2) the precise nature of the secondary—and tertiary—invaders is largely a matter of accident, dependent on the occurrence of particular bacteria in the respiratory tract of persons at the time of infection, and in the case of group outbreaks, on their occurrence in contacts; (3) the character of the resulting pneumonia, clinical and pathologic, is largely determined by the nature of the secondary invaders, whether Pfeiffer bacillus, streptococcus, pneumococcus, or other organisms; (4) there seems little doubt that the influenza virus, besides depressing the general pulmonary resistance, also acts directly on the pulmonary tissues, causing capillary necrosis, edema, and hemorrhage; (5) it seems to be true, therefore, that the fatal outcome of influenza pneumonia is determined partly by the degree to which the influenza virus depresses local and general pulmonary resistance, and partly by the virulence and nature of the bacteria which invade the tissues in the wake of the specific virus” (*6*).

## Hypothesis

We endorse a sequential-infection hypothesis. This hypothesis is consistent with the known epidemiologic and clinical characteristics of the 1918–19 influenza pandemic, reflects the consensus views of firsthand observers and contemporaneous experts, and incorporates current knowledge regarding the effects of influenza on physical and immune respiratory tract defenses and physiologic interactions between influenza and respiratory bacteria (*12*,*13*,*34*–*36*).

A novel strain of influenza spread rapidly throughout the world in 1918. For most patients, infection with the virus was clinically expressed as an “influenza-like illness” that was transiently debilitating but rarely fatal. In addition, however, the virus induced aberrant immune responses, including excessive and prolonged production of interferons, proinflammatory cytokines, and chemokines, particularly among young adults (*34*). The pathophysiologic effects included inflammation and destruction of respiratory epithelium; immune cell infiltration of lung tissue with edema and hemorrhage; and ultimately, degradation or destruction of virtually all physical and immune defenses of the lower respiratory tract (*34*). Increased susceptibility of the lower respiratory tract enabled invasion by preexisting or newly acquired colonizing strains of bacteria (*12*,*35*–*38*). The synergistic effects of infection with the virus, aberrant immune responses to the virus, and secondary opportunistic bacterial pneumonias were severe and often fatal.

Finally, for brief periods and to varying degrees, affected hosts became “cloud adults” who increased the aerosolization of colonizing strains of bacteria, particularly pneumococci, hemolytic streptococci, *H. influenzae,* and *S. aureus* (*39*). For several days during local epidemics—particularly in crowded settings such as hospital wards, military camps, troop ships, and mines—some persons were immunologically susceptible to, infected with, or recovering from infections with influenza virus. Persons with active infections were aerosolizing the bacteria that colonized their noses and throats, while others—often, in the same “breathing spaces”—were profoundly susceptible to invasion of and rapid spread through their lungs by their own or others’ colonizing bacteria.

## Implications

Why is it important to determine the major pathophysiologic pathways that led to deaths during the 1918–19 influenza pandemic? After all, the effective prevention and treatment of influenza infections during a future pandemic would prevent all secondary effects, including opportunistic bacterial pneumonias. Yet concerns exist that an effective strain-specific vaccine and effective antiviral drugs may not be produced and distributed to all at-risk populations in time to mitigate the effects of the next pandemic. In the absence of an effective influenza vaccine and antiviral drugs, circumstances during a modern influenza pandemic could resemble those in 1918–19, with the notable exception of the availability of bacterial vaccines and antibacterial drugs. The exclusive focus on the prevention and treatment of a novel strain of influenza virus is risky because it unnecessarily limits options and opportunities for other potentially effective prevention and treatment methods, especially in medically underserved populations in less-developed countries.

We suggest that preparations for the next influenza pandemic should focus on more than preventing and treating influenza virus infections. A modified influenza pandemic plan might include the following components: 1) Before a pandemic, expand indications for and decrease barriers to receipt of vaccination against *S. pneumoniae* (*36*–*38*,*40*). 2) During a pandemic, in communities not yet affected, universally vaccinate with a safe and effective strain-specific influenza vaccine, if available. 3) During local epidemics, treat all serious clinical cases with an antibacterial agent that is effective against *S. pneumoniae*, *S. pyogenes, H. influenzae, and S. aureus (*including methicillin-resistant *S. aureus*); isolate patients with clinical cases from other patients and as many others as possible (*35**,**37**–**39*). 4) Conduct pandemic-related surveillance that tracks the incidence, nature (e.g., species, affected sites, antimicrobial drug sensitivities), and outcomes of bacterial infections that complicate influenza cases.

Given highly variable colonization and drug-sensitivity patterns across populations and locations, stockpiles of antibacterial drugs should be tailored to their intended uses. Plans for providing medical care should include evidence-based triage and treatment algorithms and home-care treatment guidelines (including prepackaged antiviral and antibacterial drugs) to minimize hospitalizations and maximize home care. Perhaps most important, pandemic-related research activities (including laboratory animal studies, statistical models, and clinical trials) should elucidate the determinants and effects of bacterial pneumonias that occur secondary to influenza. Ultimately, research activities should determine the most effective uses of antibacterial drugs and bacterial vaccines (e.g., indications, agents, doses, and timing for prophylaxis and treatment) in preparation for and during pandemic influenza, particularly for medically underserved and other high-risk populations.
